# Radical trachelectomy: The first step of fertility preservation in young women with cervical cancer (Review)

**DOI:** 10.3892/or.2013.2736

**Published:** 2013-09-19

**Authors:** SALVATORE GIZZO, EMANUELE ANCONA, CARLO SACCARDI, TITO SILVIO PATRELLI, ROBERTO BERRETTA, OMAR ANIS, MARCO NOVENTA, ANNA BERTOCCO, SIMONE FAGHERAZZI, MICHELA LONGONE, LUCIA VENDEMIATI, DONATO D’ANTONA, GIOVANNI BATTISTA NARDELLI

**Affiliations:** 1 Department of Women’s and Children’s Health, University of Padua, Padua, Italy; 2 Department of Surgical Sciences, University of Parma, Parma, Italy

**Keywords:** radical trachelectomy, cervical cancer, young women, future pregnancy desire, subsequent fertility rat

## Abstract

Radical trachelectomy (RT) can be performed vaginally or abdominally (laparotomic, laparoscopic or robotic). The aim of this systematic review was to compare all techniques in terms of surgical complications, disease recurrence and subsequent fertility/pregnancy outcomes. A total of 1293 RTs were analyzed (FIGO-stage: IA1–IIA). The most frequent surgical complications do not differ from the ones of radical hysterectomy. The recurrence risk is approximately 3% (range 0–16.8%). The majority of women conceive spontaneously: 284 pregnancies with 173 live births. The most frequent pregnancy complication was miscarriage and chorioamnionitis. RT appears to be a safe option for eligible women who intend to maintain their future pregnancy desire.

## 1. Introduction

Cervical cancer is the third most commonly diagnosed cancer and the fourth leading cause of cancer-related mortality in females worldwide, accounting for 9% (529,800) of all new cancer cases and 8% (275,100) of all cancer deaths among females in 2008. More than 85% of these cases and deaths occur in developing countries. The disproportionately high burden of cervical cancer in developing countries and elsewhere in medically underserved populations is largely due to a lack of screening that allows detection of pre-cancerous and early-stage cervical cancer; the health care infrastructure in these countries does not support Papanicolaou testing or other types of screening tests (1).

Screening programs, especially in developed countries, allow for the detection of cervical cancer in young women, particularly in early stages, thereby increasing the number of patients that are completely cured. Currently, in cases of cervical cancer at FIGO stage IA2 and more, the adequate treatment is based on radical surgery with or without subsequent radiotherapy, according to the presence or absence of adjunctive risk factors (i.e. lymph vascular invasion, grading).

However, radical surgery and radiotherapy often do not spare fertility and both methods can lead to psychosexual dysfunction and decreased quality of life. Furthermore, infertility increases the frequency of depression, stress and sexual dysfunction (2). The increase of cancer detection in younger patients who plan on a future pregnancy has recently led oncologic gynecological surgeons to revise the radical surgical approaches in order to preserve the fertility (ovaries and corpus uteri) without increasing the risk of recurrence and mortality (2).

Radical vaginal trachelectomy (RVT) is a fertility-sparing technique first described by Daniel Dargent in 1994, involving the removal of the cervix, the parametrium, and cuff of vagina, while maintaining the patient’s uterine fundus and adnexae. This procedure, in combination with a laparoscopic pelvic lymphadenectomy, is the most common and accepted fertility-sparing procedure for early cervical cancer (3). Although RVT associated with laparoscopic pelvic lymphadenectomy is the most used surgical procedure, radical trachelectomy (RT) may be performed either abdominally or vaginally (laparoscopic or robotic) (4–6).

Independent of the surgical approach employed, all patients eligible for fertility-sparing surgery must conform to universal selection criteria. The ideal candidates are women in whom the tumor size is small and is confined to the cervix (no evidence of direct spread to either the parametrium or uterine corpus), no previous evidence of infertility, clinical stage between IA1 (with LVSI lymphovascular space involvement) and IB1 (tumor size <2 cm), no diagnosis of small-cell carcinoma (with or without neuroendocrine component) or sarcoma, limited endocervical involvement, no suspicion of pelvic lymph node involvement and no deep stromal invasion (>10 mm) (5).

The aim of the present systematic review was to compare all described techniques performed in patients with cervical cancer eligible for fertility-sparing surgery, in terms of surgical complications, disease recurrence rate, subsequent fertility rate and obstetrical outcomes.

## 2. Data sources

A systematic literature search was conducted in the electronic databases MEDLINE, EMBASE Sciencedirect and the Cochrane Library in the interval time between January 2000 and December 2012. We only considered articles in English. All original descriptions, case series, retrospective evaluations and review articles of women who had been treated with vaginal and abdominal (laparotomic, laparoscopic and robotic) trachelectomy were analyzed. Key search terms included trachelectomy in combination with cervical cancer, fertility, fertility-sparing surgery, radical, abdominal, vaginal, laparoscopic, robotic, pregnancy, fertility preservation, disease free survival.

A manual search of reference lists of included studies and review articles was successively performed. References of the retrieved articles were searched to identify any articles not found in the initial search. Considered outcomes were: complications of procedure, cancer recurrence rate, subsequent fertility rate and pregnancy complications in relation to surgical technique performed. We excluded studies providing ambiguous or insufficient data on procedure or obstetrical outcomes regarding short- and long-term complications, efficacy and impact on future pregnancies.

## 3. Available methods

The universally accepted optimal treatment with intention to preserve fertility in case of early-stage cervical cancer (IA1 with LVSI to IB1 with tumor size <2 cm) requires RT and pelvic lymphadenectomy. RT can be performed vaginally or abdominally (using the laparotomic, laparoscopic or robotic techniques); moreover, pelvic lymphadenectomy is performed by the laparotomic approach only in case of laparotomic trachelectomy, whereas it is carried out laparoscopically or robotically in all other cases.

Surgical approach with fertility-sparing intent does not require any different technique for pelvic lymphadenectomy compared to standard treatment for cervical cancer. Trachelectomy spares adnexae and corpus uteri. Concerning vaginal trachelectomy, it is necessary to perform a circumferential incision in the upper vagina. The anterior and posterior vaginal mucosa is grasped with Chrobak forceps. The supracervical ligament is cut and the bladder base is mobilized. Posteriorly, the pouch of Douglas is opened and the pararectal spaces are exposed. The uterosacral ligaments are then divided. The vesicovaginal ligaments are then identified and the paravesical spaces are entered laterally. At this point, the ureters are identified by palpation of the knee of the ureter, and then the vesicovaginal ligaments are separated from the cervix. The arch of the uterine artery is identified and right-angle forceps are passed through the paraisthmic window immediately below the uterine artery to define the upper limit of the cardinal ligaments. The cardinal ligaments are then divided. A Prolene suture or an Ethibond suture can be used to perform the cerclage. The uterus is then reattached to the upper vaginal cuff (6).

In case of laparotomic trachelectomy, the paravesical and pararectal spaces are developed and the bladder is dissected caudal to the mid-vagina. The round ligaments are divided, but the infundibulopelvic ligaments with the ovarian blood supply are kept intact. The uterine vessels are legated and divided at their origin from the internal iliac vessels. The parametria are mobilized medially and a complete ureterolyis is performed similar to a Piver III radical hysterectomy. The posterior cul-de-sac peritoneum is incised and the uterosacral ligaments are divided. A vaginal cylinder can be used such that the desired length of vaginectomy is performed and the specimen is separated from the vagina. The lower uterine segment is estimated and clamps are placed at the level of the internal os. The procedure is completed by separating the fundus from the isthmus. The uterus-vagina reconstruction is similar to the vaginal approach (7).

The laparoscopic and robotic approaches differ from laparotomic only by the access route used. Laparoscopy requires a camera insertion through the umbilicus and three operative trocars are placed under direct vision; two 5-mm ancillary trocars are inserted in the lower quadrants lateral to the epigastric vessels, and a 10-mm trocar is placed suprapubically in the midline. The robotic approach, instead, requires four trocars: a 12-mm transumbilical optical trocar, two 8-mm robotic trocars and a 10-mm assistant trocar (4–8). In performing RT, the surgical approach is often related to surgeon preference and their level of expertise.

All the surgical options described are developed in order to increase the chance of disease-free tissue (next to the uterine isthmus) of at least 1 cm from the resection margin; in the case of intra-operatory microscopic diagnosis of isthmus cancer invasion, it is necessary to perform a radical hysterectomy. Once adequate margins are confirmed, it is recommended to perform a cervico-isthmic cerclage in all cases after trachelectomy. A cerclage can always be performed at the time of pregnancy.

## 4. Results

In the interval time considered, on the basis of our key-search, >342 articles were available in the scientific database literature, but only 28 met our selection criteria. A total of 1293 RTs were analyzed. The FIGO stage reported ranges between IA1 to IIA.

### Surgical outcomes: intra- and post-operative complications

The largest study was conducted by Shepherd and Milliken (9) on 158 RVTs with laparoscopic bilateral pelvic lymphadenectomy. Peri-operative complications were reported in 8.5% of cases. The most frequent complication was ureteric damage incurred while performing the laparoscopic lymphadenectomy. Long-term complications occurred in 20% of patients, and included amenorrhoea, stitch expulsion or *de novo* dyspareunia.

Data regarding the intra-operative complications during vaginal radical trachelectomy (RVT) showed more frequently ureteric damage, cystotomy and vascular traumas. The vaginal approach was frequently associated with complications by post-operative dyspareunia, amenorrhoea or prolonged vaginal bleeding.

Regarding lymphadenectomy, pelvic lymphocyst with or without infection signs was often reported. Some studies showed peculiar complications such as obturator nerve injury (10), pelvic hematoma (11), isthmic stenosis (12). The introduction of the laparoscopic approach in performing RT reduced the rate of intra-operative complications; the two eligible studies (13,14) did not show complications in 43 patients treated. The robotic approach was introduced to reduce the surgical difficulty of laparoscopy. The only three eligible data series on this procedure (4,15,16) showed that the complication rates were similar to laparoscopy; the authors reported only one case of small bowel herniation and one of hemorrhage from the inferior epigastric vessels.

The laparotomic approach in performing RT can be considered a procedure with an intermediate risk in terms of intra-operative and peri-operative complications; better than RVT and worse than laparoscopic/robotic RT. Most of the studies considered did not show peri-operative complications (17,20). In 392 laparotomic RTs performed, the most frequent intra-operative complication reported was blood transfusion (10 cases) (16,21,22); only one vascular injury (external iliac artery) (22) and only one case of ureteral injury were reported (23). The most frequent post-operative complications were cervical stenosis, lymphocyst formation with or without subsequent infection, hemorrhagic cyst, cervicovaginal dehiscence and cerclage erosion (16,17–21,23,24). All detailed data are reported in Tables I–III.

### Oncological outcomes: disease recurrence

A large series of RVTs reported by Shepherd and Milliken (9) showed recurrence rates of 2.5%. Similar results were shown in the studies of Beiner and Covens (25), Diaz *et al*(26), Bernardini *et al*(27) with recurrence rates of 2.8, 2.5 and 3.6%, respectively. Other studies by Plante *et al*(28,29), Hertel *et al*(30), Marchiole *et al*(31), Dargent *et al*(32) considering, respectively, 72, 100, 118, 47 patients, showed recurrence rates >4%. Smaller studies with a less significant number of patients followed the same trend (33,34). The most frequent recurrence sites reported were the pelvic walls, locoregional lymph nodes and lung, as distant metastasis (9,25,26,28–30,32).

The laparoscopic approach showed a wider range of recurrence rates since Chen *et al*(13) did not observe recurrences while Kim *et al*(14) evidenced a recurrence rate of 7.4%. In the same way, Burnett *et al*(4) did not observe recurrence in the robotic laparoscopic approach, although they considered only 5 patients. The laparotomic approach showed a lower recurrence rate; Cibula *et al*(19), Li *et al*(21), Ungar *et al*(23) and Olawaiye *et al*(24) did not report any.

Similar to the laparoscopic approach, the most frequent recurrence sites were the pelvic walls and the para-aortic lymph nodes (18–20).

### Obstetrical outcomes: subsequent pregnancy and complication rates

RT is the only available fertility-sparing approach approved to preserve fertility in young patients with early cervical cancer. Regarding the vaginal technique, Shepherd and Milliken (9), Plante *et al*(28,29) and Dargent *et al*(32) observed pregnancy rates >50% and a live birth rate of 50, 72 and 52%, respectively. Sonoda *et al*(35) reported a 79% pregnancy rate occurring in 36% of these after assisted reproductive techniques. Live birth rate was 100%. Bernardini *et al*(27) showed a 56.4% pregnancy rate with an 82% live birth rate. Data by Diaz *et al*(26) did not provide appropriate obstetrical outcome extrapolation, since some patients had an ongoing pregnancy when the study was published.

The only two studies considering the laparoscopic approach, conducted by Chen *et al*(13) and Kim *et al*(14), reported 31 and 50% pregnancy rates while live birth rates were 0 and 33%, respectively. The difficulty in comparing obstetrical outcomes following laparoscopic vs. robotic approach is related to the limited data available. Concerning the robotic approach, the two studies performed by Burnett *et al*(4) and Nick *et al*(16) did not report pregnancies and the only other study, published recently, reported 80% pregnancy rate without specifying the live birth rate (15). On the other hand, fertility and obstetrical outcomes with regard to the laparotomic approach are widely documented.

Shepherd and Milliken (9) performed a small number of laparotomic trachelectomies (116 patients) reporting 10 pregnancies with 6 live births without specifying the number of pregnancies attempted. The remaining studies presented a wide range in terms of pregnancy rates, with the most favorable results by Olawaiye *et al*(24) (100%), Ungar *et al*(23) (60%) Pareja *et al*(22) (50%) vs. poorer results by Nishio *et al*(18) (14%) and Li *et al*(21) (3.6%). Similarly, the live birth rate ranged between 50–100% since all studies showed a rate >50%. As anticipated, in all cases the delivery was performed by classical (low vertical) caesarean section, both at term and preterm of gestation.

According to the results obtained by Shepherd and Milliken (9) (28% of chorioamnionitis with a 14% deliveries before 32 weeks) the most frequent pregnancy complications reported were miscarriage (34%), followed by chorioamnionitis with or without rupture of membranes (15.5%).

## 5. Discussion

The major difficulty in comparing the different surgical techniques by literature review was finding articles with clear, suitable and complete descriptions of each technique. In particular, a large number of the published articles did not present a proper match between oncological and obstetrical data, since they focused on one aspect only. Moreover, the difficulty increased when attempting to compare the same outcomes for the different techniques.

In the past years, fertility-sparing surgery has become a more concrete option for young patients with early-stage cervical cancer (FIGO stages IA2–2B) (2,5–7). The first surgeon to propose the feasibility of fertility-sparing treatment in this cohort of patients was Dargent in 1995 (3).

The first report of 25 RTs at the Society of Gynecologic Oncologists was met with a lot of scepticism, but as more data were obtained, the procedure began to gain acceptance in the gynecologic oncologic community. This, coupled with the finding that nearly half of women under 40 years of age undergoing radical hysterectomy for stage I cervical cancer may be eligible for RT, has made this procedure attractive to many gynecologic oncologists.

Technically, the RVT, preceded by a laparoscopic bilateral pelvic lymphadenectomy, is performed by dividing the uterus proximal to the cervical isthmus and suturing the uterus to the vagina. Intra-operative frozen sections should be used on both the endocervical margin and nodal tissue, completing radical hysterectomy if tumor extends to within 5 mm of the margin (3). The final success of fertility-sparing surgery is strictly related to the pre-operative evaluation process, which is the more critical step in decision making of feasibility.

The small size of the tumor is the first criterion for eligible patient selection; moreover, the tumor has to be confined to the cervix with no evidence of direct spread to either the parametrium or uterine corpus. The risk of nodal metastasis should also be low in these patients. The second, but not less important criterion in patient selection, is the pre-operatory definition of the subsequent pregnancy desire. In fact, the preservation of corpus uteri may make sense in order to achieve a future pregnancy. Alternatively, hormonal status could be preserved from damages of primary or adjuvant pelvic radiotherapy by ovarian transposition (36) or transplantation (37,38).

The real target of fertility-sparing surgery should be the hormonal status preservation in combination with the corpus uteri sparing, since the major concern is related to the possibility of a future pregnancy. In order to avoid it, neoadjuvant chemotherapy is proposed to remove possible ovarian micro-metastasis and/or to downstage ‘bulky’ cervical cancer. Regarding this, Vercellino *et al*(33) reported outcomes for women with large cervical lesions (FIGO IB1 >2 cm and IB2) for whom neoadjuvant chemotherapy and fertility-sparing surgery were considered. The authors concluded that pre-treatment nodal assessment could identify a high-risk subgroup for whom fertility-sparing treatment should be avoided, since they have a significant risk of recurrence even after aggressive multi-modality treatment. Despite these legitimate doubts, two of the largest series demonstrated that the oncologic safety of RT performed vaginally without neoadjuvant chemotherapy is comparable to oncologic outcomes after radical hysterectomy.

Hertel *et al*(30) reported data on a multicentric perspective trial from the German Association of Gynaecologic Oncologists; 100 patients who met strict entry criteria were treated with RVT and pelvic lymphadenectomy. After a median follow-up of 29 months (range, 1–128 months), the recurrence rate was 4%. Shepherd and Milliken (9) also reported their series of 158 consecutive patients treated with RVT and pelvic lymphadenectomy with a 2.5% of recurrence rate during a 45-month follow up. According to the importance of accurate patient selection, the author reported the 9% of conversion of RVT on radical hysterectomy was due to detection of adverse prognostic factors, such as positive lymph nodes detected unexpectedly or impossibility of obtaining proper excision margins.

If the oncologic criteria are satisfied, all described approaches, with slight differences, are safe in terms of peri-operative and post-operative complications.

RVT is described as a safe surgical procedure to preserve fertility (25,30,31) although the surgeons should be trained in radical vaginal procedures which, nowadays, are an infrequent step in oncologic training programs. The surgeons should also be trained in the abdominal approach and skilled in multi-organ injury repair, since the rates of conversion to open surgery or secondary surgery for intra-operative or post-operative complications ranged between 0 and 9% (28,29,32). The most frequent injury reported involved the urinary tract or bowel: all authors reported that the repair was performed during primary surgery without negative sequelae for the patient (9,28,29,32).

Compared with RVT, radical abdominal trachelectomy is practiced by relatively few surgeons. The abdominal approach, as a first option, should be performed in patients with distorted vaginal anatomy, larger lesions or in cases of nulliparous patients with little lumen of vagina. The laparotomic approach has some advantages compared to the laparoscopic one: the decrease of the cost of equipment and necessity of training, the reduction of intra-operative complications since the gynecologic oncologists are more familiar with laparotomy.

The laparotomic approach should be encouraged on the basis of the positive oncologic and obstetrics outcome following the procedure (21). This could be considered anachronistic since in the era of mini-invasive surgery both the laparoscopic and robotic approaches showed an increasing affirmation related to their best surgical and oncologic outcome. Therefore, after adequate surgeon skill acquisition, laparoscopic and robotic approaches should not be excluded from this field. From the first proposal by Dargent, laparoscopy became the gold standard in performing pelvic lymphadenectomy before vaginal RT.

At present, laparoscopy and robotic surgery represent a noteworthy alternative approach in performing RT and pelvic lymphadenectomy. Burnett *et al*(4) proposed the first series of robotic surgery with the intent to demonstrate that this approach may circumvent some of the disadvantages related to either vaginal or laparotomic/laparoscopic RT. The advantages related to the robotic system, recently confirmed by Persson *et al*(15) are summarized in the improved visualization due to three-dimensional optics, the improved tissue manipulation due to flexibility of the instruments, the improved fine dissection, the minimal invasion resulting in decreased adjacent tissue trauma and adhesions, the diminished post-operative pain and hospitalization.

Despite the optimal surgical results, caution in proposing laparoscopy and the robotic approaches is due to the limited data regarding subsequent obstetrical outcomes. The main problem is related to the difficulty in restoring the junction between upper vagina and corpus uteri. This surgical time is considered fundamental to reduce certain pregnancy complications such as preterm delivery due to pPROM and chorioamnionitis.

The most accepted theory links the high incidence of preterm delivery and increased risk of infection to the absence of cervical barrier function, the absence of cervical mucus plug and non physiological competence obtained by isthmic cerclage (27). An adjunctive risk factor for preterm delivery is considered the uterine vessel damage which causes decreased uterine blood flow (14,27). Particularly during the abdominal approach, both uterine arteries are usually divided to dissect the cardinal ligaments and the ureters from the uterine arteries while during the vaginal approach, both uterine arteries are usually preserved. Burnett *et al*(4) proposed resolving these problems through the robotic approach; the Da Vinci system allows dissecting the uterine vessels from the hypogastric artery to the corpus. At the corpus, the ascending branch of the uterine artery can be successfully maintained with cauterization of the descending branches to the cervix.

## 6. Conclusion

With the earlier detection of invasive cervical cancer, fertility preservation is increasingly needed. At present, since childbearing is postponed, it is more common to detect cervical cancer in women who have not already been pregnant or desire a future pregnancy. Fertility preservation with conservation of the uterus after an RT seems to be a safe and realistic option for well-motivated women that wish to maintain their fertility, since recurrence rates are acceptably low, but higher than traditional radical hysterectomy.

The review of the literature indicates that pregnancies are clearly possible after an RT for early-stage cervical cancer, and the majority of women will conceive spontaneously. However there is a higher incidence of preterm deliveries, miscarriages, chorioamnionitis and pPROM. Although the few aspects related to obstetrical outcome should be cleared and improved, the 284 pregnancies with 173 children born after surgical fertility-sparing surgery are encouraging.

Multicentric perspective randomized studies and multidisciplinary approaches are necessary to define the most favorable and appropriate management both in the definition of strict eligibility criteria and surgical approach. Obstetrical management will consequently improve to the increasing number of pregnancies achieved after this modulated radical surgery.

## Figures and Tables

**Table I:** Systematic review of radical vaginal trachelectomy: surgical outcome, oncological outcome and subsequent obstetrical outcome.

Author/(Refs.)	No. of patients	FIGO stage (No.)	Peri-operative complications	Post-operative complications	Recurrence site and patient no.	Recurrence rate (%)	Attempted pregnancies	No. of pregnancies^a^	Live births	No. of delivery <32 weeks	Type and no. of pregnancy complications	Note
Shepherd and Milliken (9)	158	IB1 (152)	χ,3; Π, 3	Ψ, 8; γ, 5	Vaginal vault, 1	2.5	31	88	44	10	PROM and chorioamnionitis, 25	Ongoing pregnancies, 7
		IIA (2)	Ǿ,2; Ω, 1	T, 10; α:3	Left paracervical tissue, 1						Stillbirths, 1	
		IA2 (3)	∑, 1	ζ, 2; δ, 1	Left pelvic side wall, 1						Miscarriages, 31	
			ɛ1; β, 1		Para-aortic region, 1						Ectopic pregnancy, 1	
Dargent *et al*(32)	47	IA1 (5);	∑, 1	NA	Lateropelvic, 1	4.3		25	13	0	Miscarriages, 11	-
		IA2 (13)	H, 2		Distant, 1							
		IB (25)										
		IA2 (1)										
		IIB (3)										
Sonoda *et al*(35)	36	IA1 (8)	β,2; Ǿ, 1	-	-	2.8	14	11	11		Miscarriages, 1	IVF, 3
		IA2 (7)	δ, 2; ϰ, 1								Cervical shortenings, 1	IUI, 1
		IB1 (28)										Voluntary interruptions, 2
Plante *et al*(28)	72	IA1 (4)	Ω, 3	γ, 13; λ, 2	Left parametrium, 1	4.2	31	50	36	3	Miscarriages, 10	Ongoing pregnancies, 2
		IA2 (23)	Ǿ, 1	μ, 10; π, 9	Lung + liver metastasis, 1						Therapeutic abortions, 2	
		IB1 (43)	∑, 1	β, 9; δ, 3	Mesosigmoid, 1							
		IIA (2)		ϰ :1							p-PROM, 1	
Hertel *et al*(30)	100	IA1 (18)	β, 1;	δ, 1; κ, 4	Residual of cervix, 1	4.0	NA	18	12	1	-	Ongoing pregnancies, 3
		IA2 (21)		Ψ, 8; Ǿ, 1	Uterus, 2							
		IB1 (69)			Pelvic side wall, 1							Voluntary interruptions, 2
Diaz *et al*(26)	40	IB1 (40)	-	Ψ, 1	LN-vascular invasion, 1	2.5		9	4	0	Miscarriages, 1	Voluntary interruptions, 2
											PROM, 1	Ongoing pregnancies, 2
Marchiole *et al*(31)	118	I–IIA (n.r.)	ϱ, 1	Ǿ, 9; π, 3	Latero-pelvic, 2;	5.9	-	NA	NA	NA	NA	-
		χ, 1	β, 6; Ψ, 2		Lung, 3;							
		Ω, 1			Surrenal glands, 1							
					Lombo-aortic LN, 4;							
					Parametria, 2;3							
					Liver, 1							
Alexander-Sefre *et al*(12)	29	NA	Ω, 2,	α, 2; T, 4	0	0.0	-	-	-	-	-	-
			γ, 1;	Ψ, 3; δ, 1								
			μ, 1;	β,1; ζ, 6								
Beiner and Covens (25)	90	IA–IB (n.r.)	∑, 1		Upper abdomen, 1	3.6	-	-	-	-	-	-
			Ω, 1		Para-aortic LN, 2							
			χ, 1		Supraclavicular LN, 1							
					Lung, 1							
					Pelvic wall, 1		-	-	-	-	-	
Pahisa *et al*(34)	15	IB1 (15)	ϱ, 1		Presacral mass, 1;							Ongoing pregnancies, 2
			π, 1		Residual of the cervix, 1;	13.3		3	1	-	-	
Burnett *et al (*10)	21	IA2–IIA	κ, 3	β, 1	0	0.0		3	2	1	PROM and chorioamnionitis, 1;	
			π, 1						2	1		-
Schlaerth *et al*(11)	10	IA2 (8);		Ψ, 2	0	0.0		4	2	3	p-PROM, 1	-
		IB (2)		π, 1							Fetal death, 1	
Bernardini *et al*(27)	80	IA (n.r.)										
		IB (n.r.)	-	-	-	1.3	39	22	18	3	p-PROM, 4;	IVF, 3
											PROM, 2;	IUI, 3
											HELLP, 1;	
											Placenta previa, 1;	
Vercellino *et al*(33)	12	IB1 (7)	No	Ψ, 1	Locoregionally, 2	16.7	-	1	1	0	No	
		IB2 (5)		δ, 1	Distant, 1							

χ, ureteric damage; Π, uterine perforation; Ǿ, bleeding; Ω, vascular injury; n.r., not reported; T, cerclage expulsion; Ψ, isthmic stenosis; ∑, cystotomy; α, amenorrhoea; β, lymphocyst; γ, bladder hypotonia; δ, lymphedema; ɛ, haemorrhage; ζ, dyspareunia; η, pelvic collection; κ, nerve injury; λ, urinary tract infection; μ, vulvar edema; π, hematoma; ϱ, bladder injury; LN, lymph nodes; NA, not available; RVT, radical vaginal trachelectomy; IVF, *in vitro* fertilization; IUI, intrauterine insemination; PROM, premature rupture of membranes; p-PROM, preterm premature rupture of membranes; HELLP, hemolysis elevated liver enzymes low platelet count.

a Some patients had more than one pregnancy.

**Table II:** Systematic review of radical trachelectomy by robotic and laparoscopic technique: surgical outcome, oncological outcome and subsequent obstetrical outcome.

Author/(Refs.)	No. of patients	FIGO stage (No.)	Peri-operative complications	Post-operative complications	Recurrence site and patient no.	Recurrence rate (%)	Attempted pregnancies	No. of pregnancies^a^	Live births	No. of delivery <32 weeks	Type and no. of pregnancy complications	Note
Chen *et al*(13)	16	IA1 (3);	No	α, 1	0	0.0	NA	5	0	4	Miscarriages, 2;	Ongoing pregnancies, 1 IVF, 1
		IA2 (7);									p-PROM and amniositis, 1	
		IB1 (6);										
Kim *et al*(14)	27	IB1 (26);	No	σ, 3;	Fornix, 1	7.4	6	3	1	2	Miscarriages, 2	LEEP before LRT, 15
		IIA (1);		τ, 8								
Burnett *et al*(4)^b^	5	IB1 (5);		υ, 1;	0	0.0		0	0	0		
				Ω, 1								
Persson *et al*(15)^b^	13	IA1 (4);			0	0	5	4				
		IA2 (5);										
		IB1 (4);										
					0	0	5	4				
Nick *et al*(16)	12	IA1 (3);	-	γ, 1	0	0	11^c^	0	0	0		
		IA2 (4);		T, 0								
		IB1 (5);		α, 1								

Post-operative complications: T, cerclage expulsion; γ, bladder hypotonia; σ, dysmenorrhoea; τ, decreased menstrual flow; υ, herniation of the small bowel; Ω, vascular injury; α, amenorrhoea. NA, not available; IVF, *in vitro* fertilization; IUI, intrauterine insemination; LEEP, loop electrosurgical excision procedure; PROM, premature rupture of membranes.

a Some patients had more than one pregnancy,

b robotic technique,

c data considering both robotic and open surgery.

**Table III:** Systematic review of laparotomic trachelectomy: surgical outcome, oncological outcome and subsequent obstetrical outcome.

Author/(Refs.)	No. of patients	FIGO stage (No.)	Peri-operative complications	Post-operative complications	Recurrence site and patient no.	Recurrence rate (%)	Attempted pregnancies	No. of pregnancies^a^	Live births	No. of delivery <32 weeks	Type and no. of pregnancy complications	Note
Shepherd and Milliken (9)	116	IB1 (n.r.)	NA	NA	n.r.	1.7		10	6	2	NA	
		IIA (n.r)										
		A2 (n.r.)										
Abu-Rustum *et al*(17)	19	IB1 (22)		Ψ, 4; β, 2	0	0		NA	NA	NA	NA	Post-trachelectomy chemoradiation, 4
				T, 1; δ, 1								
				α, 1								
Olawaiye *et al*(24)	10	IA1 (1)	φ, 1	Ψ, 2	0	0	3	4	3	0	No	IVF, 2;
		IA2 (3)		T, 2								IUI, 1
		IB1 (5)										Ongoing pregnancies, 1
		IIA (1)										
Nishio *et al*(18)	61	IA1 (4)		Ψ, 1; α, 5	Cervical stump, 1	9.8	29	4	4	2	No	AIH, 1
		IA2 (8)		ɛ, 1; β, 9	Fallopian tube, 1							IVF, 1
		IB1 (49)			Bladder surface, 1							
					Common iliac artery LN, 2							
					Pelvic sidewall, 2							
					Surface of sacral bone, 1							
Cibula *et al*(19)	24	IB1 (n.r.)				0		6	5	2		
		IA2 (n.r.)										
Ungar *et al*(23)	30	IA (10)	χ, 1		0	0	5	3	2		First trimester	IVF pregnancy, 1
		IB1 (15)									Miscarriage, 1	
		IB2 (5)							;			
Li *et al*(21)	62	IA1 (16)	Ω, 4	Ψ, 5	0	0	56	2	1	0	No	Lost ovarian function after adjuvant chemoradiation, 3
		IA2 (7)		γ, 1								
		IB1 (36)		β, 2								
												Ongoing pregnancies, 1
Saso *et al*(20)	30	IA2 (2)	No	ς, 1	Lung, 1		10	3	2	1	p-PROM and chorioamnionitis, 1	
		IB1 (25)			Uterus, 1							
		IB2 (2)			Bladder, 1							
		IIA (1)			Rectum, 1							
Pareja *et al*(22)	15	IA2 (3)	ω, 4	F, 1	0	0	6	3	3	1	PROM and chorioamnionitis, 1	
		IB1 (12)	Ω, 1	T, 2								
Nick *et al*(16)	25	IA1 (20	ω, 2	γ, 6	0	0	11^b^	3	2	1	No	
		IA2 (7)		T, 4								
		IB1 (16)		α, 7								

α, amenorrhoea; φ, pulmonary embolism; ω, intraoperative blood transfusion; γ, bladder hypotonia; T, cerclage expulsion; Ψ, isthmic stenosis; ς, haematocolpos; F, tubo-ovarian abscess; Ω, vascular injury; AIH; artificial insemination of host; NA, not available; IVF, *in vitro* fertilization; IUI, intrauterine insemination.

a Some patients had more than one pregnancy,

b data considering both robotic and open surgery.
